# Supplementary material for: TastepepAI: An artificial intelligence platform for taste peptide de novo design
Source: PLoS Comput Biol. 2025 Oct 16;21(10):e1013602. doi: 10.1371/journal.pcbi.1013602 (PMC12543283; doi:10.1371/journal.pcbi.1013602)
Supplement: S1 File — (ZIP) [file pcbi.1013602.s006.zip › Supplementary Data 2/TaPep51 HPLC.doc]

**HPLC REPORT**

| Sample: | NJP93354 TaPep51 SR-6 | | | Analyzed date: | 2024-10-10 |
| --- | --- | --- | --- | --- | --- |
| Analyst: | WJ | | | Reconstitution: | 100%DMSO |
| Lot. No.: | NJP93354-1172279 | | | | |
| Column: | 4.6×250mm,Venusil MP C18-5 | | | | |
| Solvent A | A: 0.1% Trifluoroacetic Acid in 100% Acetonitrile | | | | |
| Solvent B | B: 0.1% Trifluoroacetic Acid in 100% Water | | | | |
| Gradient: |  | A | B |  | |
|  | 0.0min | 3% | 97% |  | |
|  | 25.0min | 40% | 60% |  | |
|  | 25.1min | 100% | 0% |  | |
|  | 30.0min | Stop | |  | |
| Volume: | 5μl | | |  | |
| Wavelength: | 220nm | | |  | |
| Flow rate: | 1.0ml/min | | |  | |

────────────────────────────────────────────────────────────

Rank	Time	Conc. Area	Height

────────────────────────────────────────────────────────────

1	13.625	0.6093	14446	1256

2	13.751	1.5578	36932	3486

3	14.008	96.7000	2292543	274193

4	14.288	1.1329	26858	3236

────────────────────────────────────────────────────────────

Total	100	2370779	282171
